# A rare case of huge maxillary ameloblastoma in a 3.5 years old girl

**DOI:** 10.1016/j.ijscr.2020.06.027

**Published:** 2020-06-12

**Authors:** Mohamed El Sayed, Mohamed Touny, Nesreen Ibrahim, Zainab Al-Azzawi

**Affiliations:** a Department of Otolaryngology-Head and Neck Surgery, Faculty of Medicine, Benha University, Benha, Egypt; b Department of Oral and Maxillofacial Surgery Nasser Institute Hospital, Cairo, Egypt; c The University of Jordan, Jordan; d Smile Clinic Of Berkley, MI, USA

**Keywords:** Case report, Amelobalstoma, Maxilla, Child, Buccal pad of fat

## Abstract

Ameloblastoma is a benign locally invasive odontogenic tumor. It is the most common odontogenic tumor [1]. Usually, in the middle-aged population, it is more common in the mandible with about 5–20% occurring in the maxilla. We report a case of maxillary unicystic ameloblastoma in a 3.5-year-old girl. Which presented clinically as a large facial swelling causing severe facial deformation and pressure symptoms on the left eye. CT scan showed that the swelling extended to involve the orbital floor. The lesion was diagnosed and confirmed by incisional biopsy then was successfully managed by maxillectomy and immediate reconstruction using the buccal pad of fat. One year follow up with no further complaints and no recurrence was observed.

"Written informed consent was obtained from the patient guardians for publication of this case report and accompanying images. A copy of the written consent is available for review by the Editor-in-Chief of this journal on request”.

## Highlights

• Maxillary Ameloblastoma.
• Resection and reconstruction using buccal pad of fat.
• Healing after maxillectomy and reconstruction using buccal pad of fat.

## 1 Introduction

Ameloblastoma is a benign locally invasive neoplasm, it is derived from odontogenic epithelium [3]. It’s the most common odontogenic tumor of the oral cavity and accounts for about 1 percent of all oral tumors and 11% of all odontogenic tumors. Ameloblastomas are present over a wide age range, but rarely seen in children [3]. Most cases are diagnosed during the third and fifth decades of life. About 80% of ameloblastomas occur in the mandible with 70% arise in the molar region and ascending ramus, 20% in the premolar region, 10% in the incisor region and about 20% occur in the maxilla, mostly in the molar region and can extend to involve the maxillary antrum too [3]. According to the World Health Organization classification in 2005 ameloblastoma has been classified into four types: multicystic or conventional solid, unicystic, peripheral, and desmoplastic. The most common type is multicystic and it accounts for about 75–86% of all cases [4]. Histologically ameloblastoma can have plexiform or follicular patterns, and these patterns are described according to the arrangement of the tumor epithelium [2]. In some tumors, both patterns coexist [3].

Radiographically, ameloblastoma can appear as multilocular or unilocular radiolucency, most commonly it appears as a multiloculated radiolucency resulting in a honeycomb or as a soap bubble appearance. Roots of the teeth involved in the lesion may show varying degrees of resorption [4].

The tumor is slow-growing and in its earliest stages, it might be asymptomatic and could be discovered incidentally. But when the tumor enlarges patients start to have symptoms like pain, facial deformity, and enlargement of the jaw-bones [4].

This case report presents a case of a rare unicystic maxillary ameloblastoma in a 3.5-years old child. All work has been reported in line with the SCARE criteria [1].

## 2 Case presentation

**Fig. 1:**
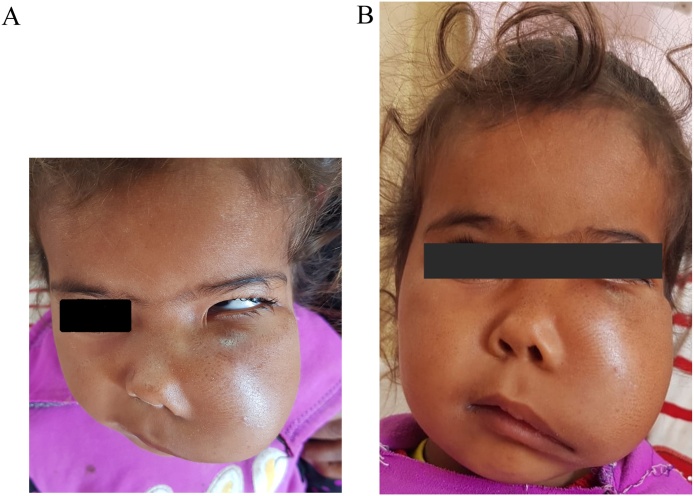
(a, b) Photograph of the patient, showing diffuse swelling at left side of face causing facial asymmetry and pressure symptoms on left eye.

A 3.5-year old girl, with no medical history, presented with swelling and pain on the left side of the face. Initially, two months back the swelling was asymptomatic, it was smaller in size, but it gradually enlarged to the present size and extended to the floor of the orbit, causing facial deformity and pain (Fig. 1). There was no history of trauma or discharge reported by the parents. The patient had pressure symptoms from the enlarged swelling.

**Fig. 2:**
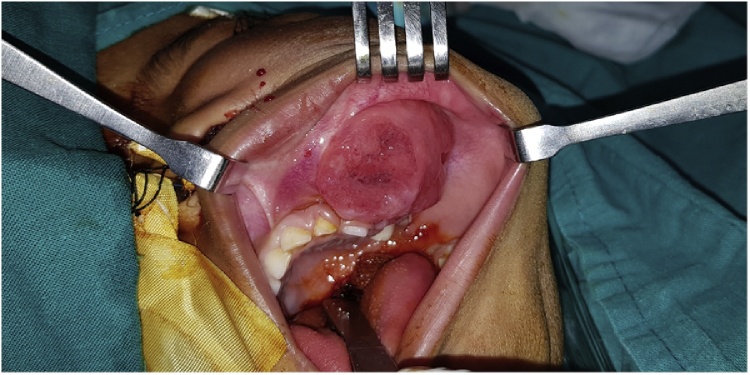
Pre-operative intra-oral view showing tumor extension.

On extraoral examination, a diffused swelling over the left side of the face measures about 7 × 7 cm, involving the maxilla and the left eye. The skin overlying the swelling was stretched, the surface was smooth, and the skin color was normal. In palpation, it was hard and painful with no local rise in temperature. Intraoral examination revealed diffuse swelling in the left maxilla, extending from the midline to the posterior molars area and expanding laterally to involve the buccal vestibule and the cheek (Fig. 2).

**Fig. 3:**
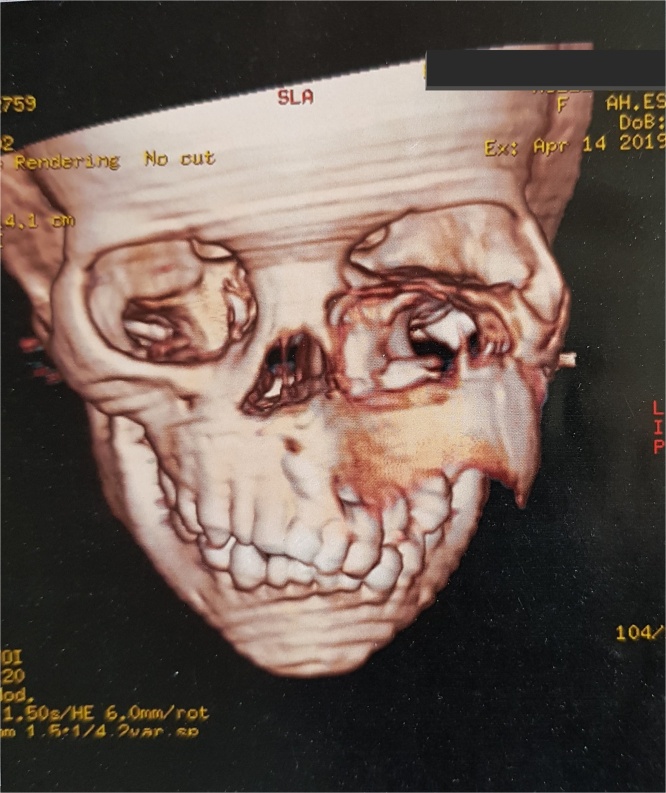
3D volume reconstructed CT showing expansile large lesion in maxilla and left orbit.

**Fig. 4:**
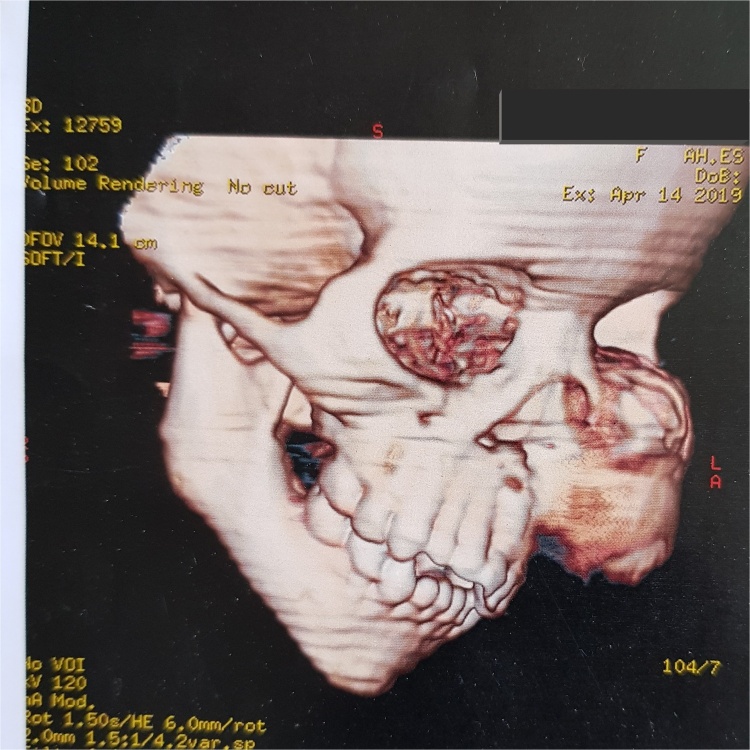
3D volume reconstructed CT showing the destruction and extension of the tumor into the left orbit.

**Fig. 5:**
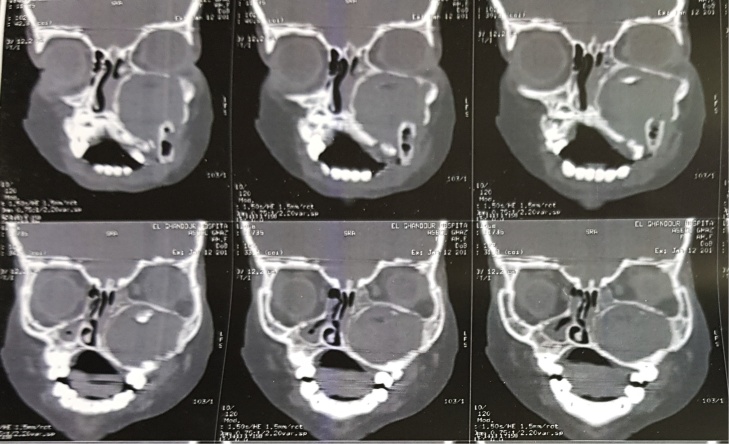
Coronal cuts demonstrating a large well-defined radiolucent expansile unilocular lesion.

**Fig. 6:**
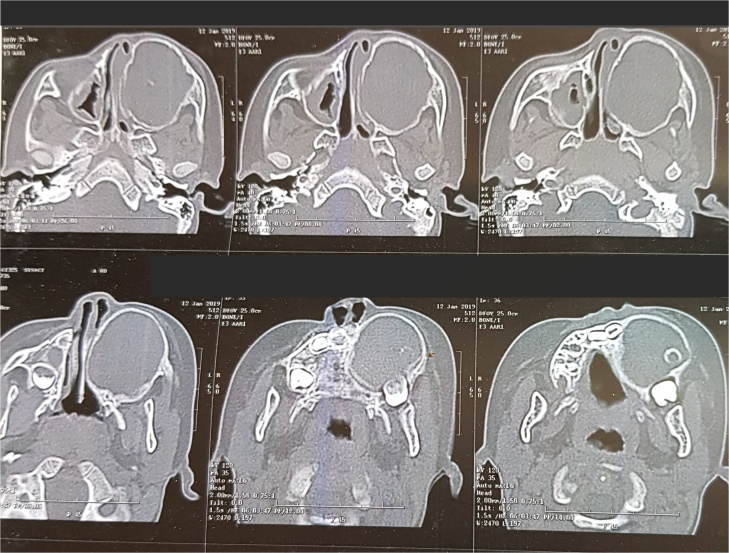
Axial cuts demonstrating a large well-defined radiolucent expansile unilocualr lesion extending posteriorly to the pterygoid plates.

**Fig. 7:**
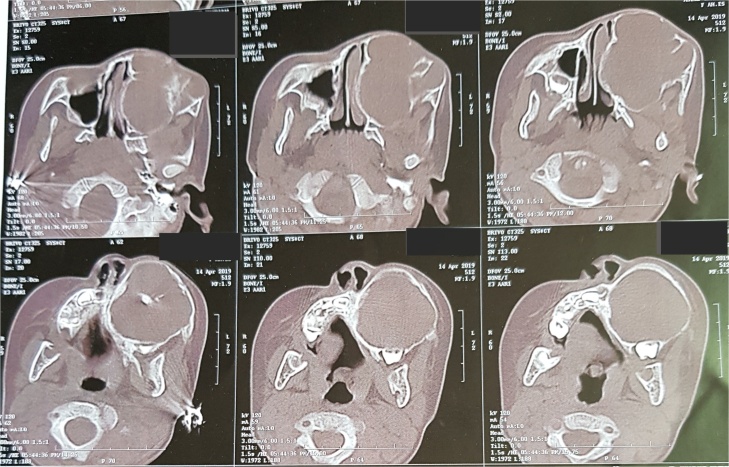
Axial cuts showing destruction of the maxilla and the extensions of the tumor causing severe disfigurement to the child’s face.

Computed tomography revealed a very large well- defined radiolucent expansile unilocular lesion in the left maxilla with perforation to the floor of the orbit (Fig. 3, Fig. 4, Fig. 5, Fig. 6, Fig. 7). Then incisional biopsy was done, small 2 pieces about 1.5 × 1.5 × 1.0 cm and 0.8 × 0.6 × 0.5 cm removed from the swelling and sent to an oral pathologist for histopathological examination. The microscopic examination of the multiple serial sections prepared from the specimen revealed strips of stratified squamous epithelium and underlying tumor tissue formed of odontogenic epithelial islands composed of non-keratinized stratified epithelium of 2–4 layers devoid of superficial keratinization with no rete ridges and with a flat interface formed by peripheral palisading of columnar cells at the basal layer with hyper-chromatic nuclei, the cells show reverse polarization away from the basement membrane with stellate reticulum-like cells, suprabasal cells composed of loosely arranged angular cells. These findings were strongly suggestive of unicystic ameloblastoma.

**Fig. 8:**
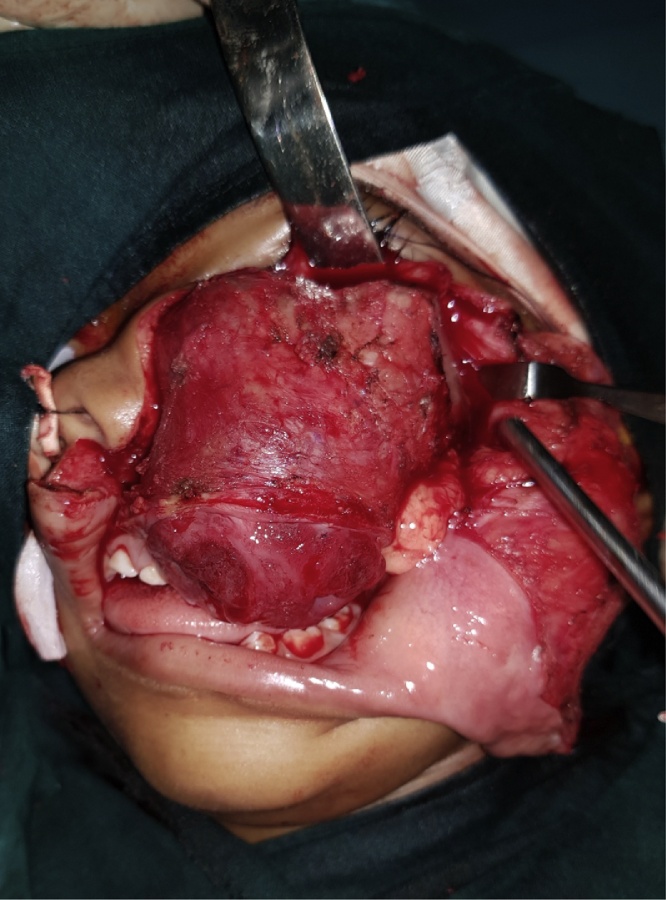
Intraoperative exposure through weber ferguson’s incison before resection.

**Fig. 9:**
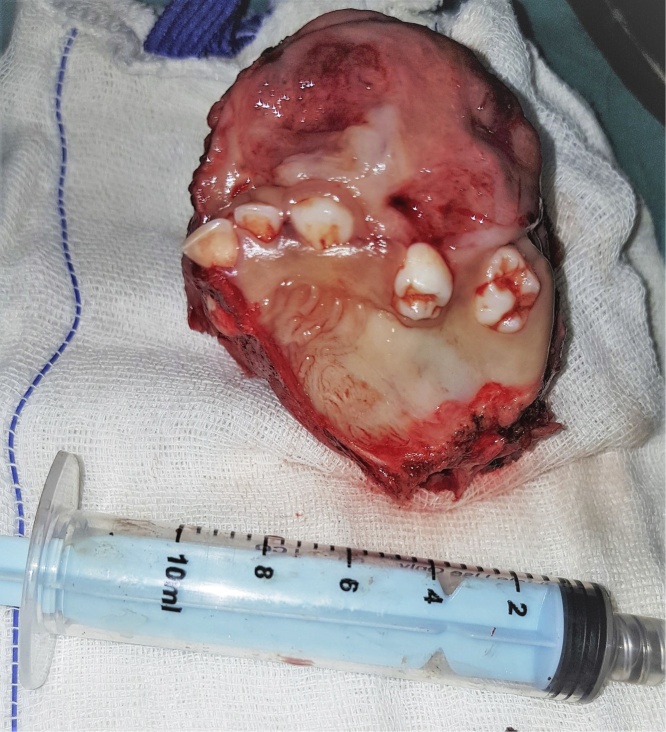
Partial maxillectomy performed with removal of the floor of orbit with 1 cm safety margins from all directions.

**Fig. 10:**
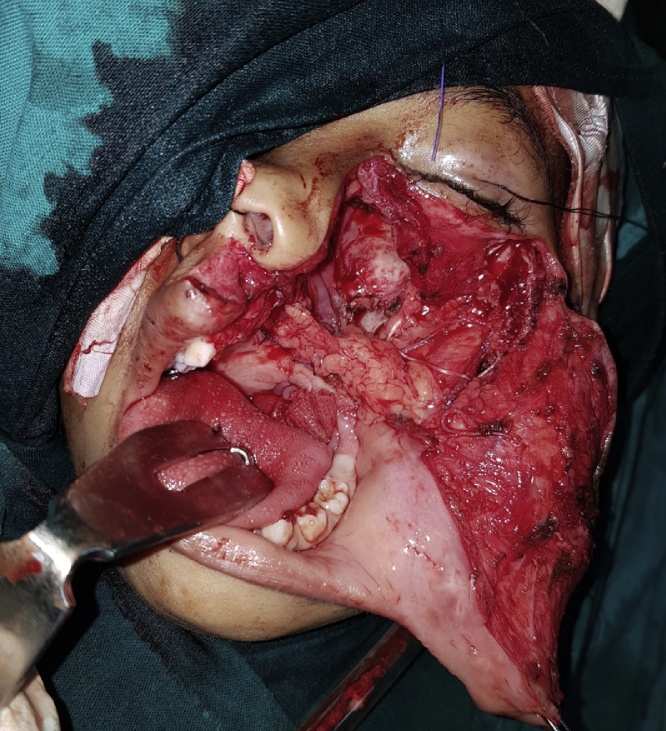
Intra-operative field after resection.

**Fig. 11:**
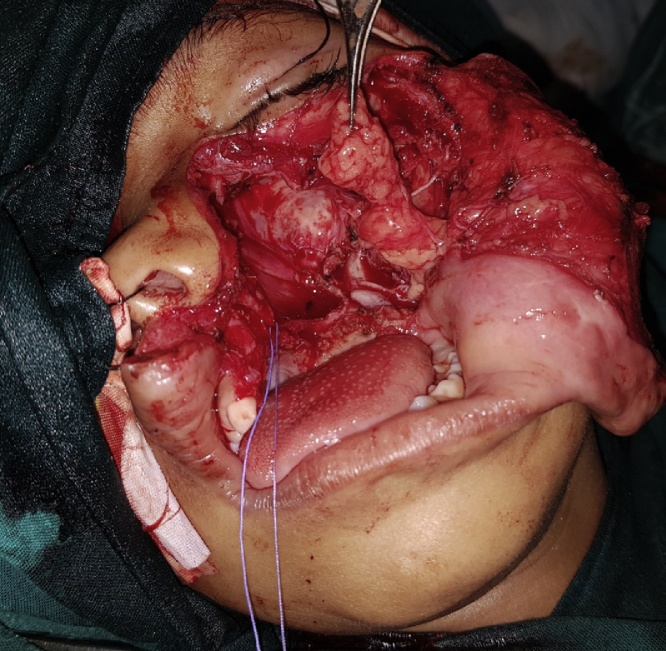
Immediate reconstruction using buccal pad of fat.

**Fig. 12:**
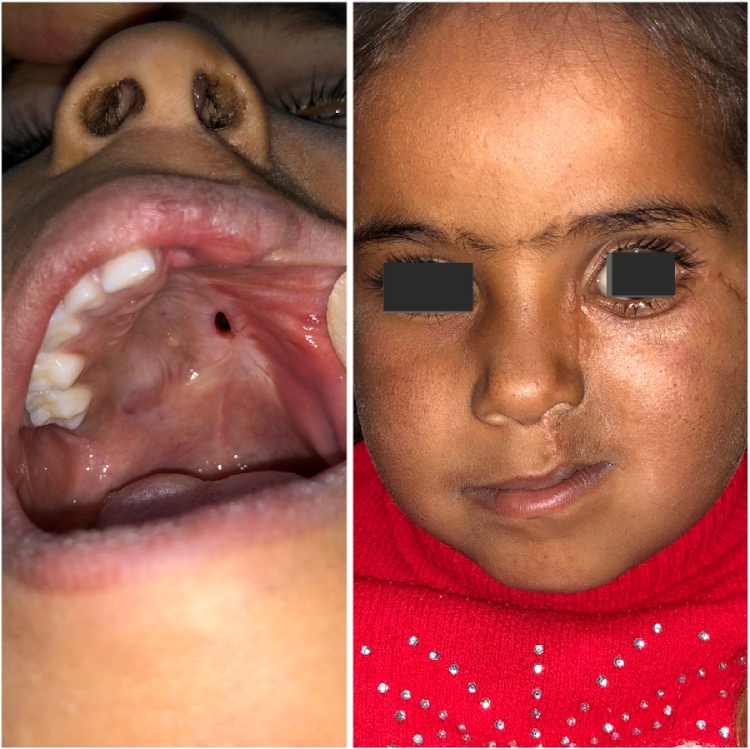
One year follow up with remaining 3 mm fistula in the palate which will be addressed later.

As the lesion was very extensive, the entire tumor has been removed using an extraoral incision (Weber Ferguson) (Fig. 8, Fig. 9) [5], After tumor exposure lesion appeared to be extending from midline till pterygoid plates on the left side and superiorly to the floor of the orbit. partial maxillectomy performed with removal of the floor of orbit with 1 cm safety margins from all directions using surgical saw and osteotomes. immediate reconstruction using the buccal pad of fat (Fig. 10, Fig. 11) [5] was done. The specimen was sent to the histopathology department and all margins were safe. The patient was followed up for one year and showed excellent healing and good functional and esthetic outcome. (Fig. 12) only 3 mm fistula remains in the palate which will be addressed later in a simple operation with no evidence of complication or recurrence.

## 3 Discussion

Ameloblastoma is a locally aggressive, epithelial odontogenic neoplasm, histologically, it resembles an enamel organ. It is non inductive which means that it fails to induce any formed calcified product such as enamel, dentin, or other materials [7].

Ameloblastoma is classified into four types: multicystic, unicystic, peripheral, and desmoplastic. The most common form is the multicystic one. This case report discusses the unicystic type. The unicystic ameloblastoma was originally described by Robinson and Martinez in 1977. Unicystic ameloblastoma in comparison to multicystic ameloblastoma occurs mostly at a younger age with a range of 10.6–23.8 years [7]. The average age of the patient at the time of initial diagnosis was 32.5 years, which is similar to that in the Chinese population in whom tumors were presented at the mean age of 32.4 years. Overall, it may be said that it is a disease involving the middle-age population in most cases [9].

If a preliminary diagnosis of ameloblastoma is made, then CT imaging is highly recommended. CT imagining can demonstrate the anatomical extent of the tumor, therefore it can detect perforation of the outer cortex and invasion into surrounding soft tissues. Accordingly, if the soft tissue invasion is extensive then Magnetic Resonance Imaging (MRI) is recommended. In a post-surgical follow-up, CT examination is crucial [8].

Incisional biopsies of large jaw cysts should be carefully examined for the presence of ameloblastomatous change, the enucleated specimens should be thoroughly sectioned to rule out mural or transmural extension. Unicystic ameloblastoma mostly is found as luminal and intraluminal forms [2].

For Cystic ameloblastoma, treatment options can range from enucleation to resection, although they are more likely to recur if enucleated [2]. In our case the lesion was very extensive, the entire maxilla with the tumor has to be removed using Weber Ferguson flap, along with reconstruction using the buccal pad of fat. The result was acceptable with one year follow up. In general, ameloblastoma should be followed indefinitely because of recurrences may be seen as long as 10–20 years after primary therapy. Ameloblastomas of the maxilla generally are more difficult to treat and manage than those of the mandible because of the anatomic relationships. In addition, the maxilla contains more cancellous bone than the mandible. Therefore, intraosseous maxillary ameloblastomas are often excised with a wider normal margin than the mandibular tumors [6].

## 4 Conclusion

This case of Unicystic Ameloblastoma was diagnosed carefully based on history and investigations. Ameloblastoma is more common in the mandible than in the maxilla, and in middle age than in children. Very rarely, we come across a case of ameloblastoma in the maxilla in a 3.5 years old child. Unicystic ameloblastoma is a tumor with a strong propensity for recurrence. Therefore, Adequate maxillectomy was performed with simultaneous reconstruction using buccal pad of fat with very acceptable results both functionally and esthetically.

## Declaration of Competing Interest

We wish to confirm that there are no known conflicts of interest associated with this publication and there has been no significant financial support for this work that could have influenced its outcome.

## Funding

No funding was received for this work.

## Ethical approval

In accordance to declaration of Helsinki Patient guardians wrote informed consent for surgical intervention and for publication.

## Consent

“Written informed consent was obtained from the patient guardians for publication of this case report and accompanying images. A copy of the written consent is available for review by the Editor-in-Chief of this journal on request”.

## Author contribution

Dr Mohamed Touny: Writing the paper. First Surgery Assistant.

Professor Mohamed El Sayed: Consultant in charge of the surgery.

Primary surgeon. Postoperative follow up of the patient.

Dr Nesreen Ibrahim: Data collection. Analysis of data. Patient follow up.

Dr Zainab Al-Azzawi: Patient follow up. Data collection. Revision of manuscript.

## Registration of research studies

NA.

## Guarantor

Dr Mohamed Touny.

Professor Mohamed El Sayed.

## Provenance and peer review

Not commissioned, externally peer-reviewed.
